# Is MS an inflammatory or primary degenerative disease?

**DOI:** 10.1007/s00702-013-1079-9

**Published:** 2013-09-06

**Authors:** Jacek Losy

**Affiliations:** 1 Department of Clinical Neuroimmunology, University School of Medicine, Poznan, Poland; 2 Neuroimmunological Unit, Institute of Experimental and Clinical Medicine, Polish Academy of Sciences, Przybyszewskiego 49, 60-355 Poznan, Poland

**Keywords:** Multiple sclerosis, Inflammation, Neurodegeneration, Cytokines, Chemokines, Autoimmunity

## Abstract

Multiple sclerosis (MS) is characterized by multiple areas of inflammation, demyelination and neurodegeneration. Multiple molecular and cellular components mediate neuroinflammation in MS. They involve: adhesion molecules, chemokines, cytokines, matalloproteases and the following cells: CD4+ T cells, CD8+ T cells, B cells, microglia and macrophages. Infiltrating Th1 CD4+ T cells secrete proinflammatory cytokines. They stimulate the release of chemokines, expression of adhesion molecules and can be factors that cause damage to the myelin sheath and axons. Chemokines stimulate integrin activation, mediate leukocyte locomotion on endothelial cells and participate in transendothelial migration. CD8+ cells can directly damage axons. B cells are involved in the production of antibodies which can participate in demyelination. B cells can also function as antigen presenting cells and contribute to T cell activation. Neuroinflammation is not only present in relapsing–remitting MS, but also in the secondary and primary progressive forms of the disease. The association between inflammation consisting of T cells, B cells, plasma cells and macrophages and axonal injury exists in MS patients including the progressive forms of the disease. The above association does not exclude the possibility that neurodegeneration can exist independently from inflammation. Very little inflammation is seen in cortical MS plaques. Anti-inflammatory therapies with different mode of action change the course of MS. Anti-inflammatory and immunomodulatory treatments are beneficial in the early relapsing stage of MS, but these treatments are ineffective in secondary progressive and primary progressive MS. In the stage of progressive MS, inflammation becomes trapped behind a closed or repaired blood–brain barrier. In such a situation current immunomodulatory, immunosuppressive or anti-inflammatory treatments might not reach this inflammatory process to exert a beneficial effect.

## Introduction

Multiple sclerosis (MS) is a chronic inflammatory disease of the central nervous system, which leads to the development of focal inflammatory lesions with secondary axonal damage. This concept has recently been challenged by several observations, suggesting that in MS neurodegeneration may occur independently of inflammation. More radical view suggests that the primary process in MS is neurodegeneration augmented by a secondary inflammatory reaction. Are there any arguments for this concept?

In contrast to white matter lesions, very little inflammation is seen in cortical MS plaques (Lassmann 2010). This could suggest that cortical demyelination can develop independently from inflammation. However, it is also evident that cortical demyelination occurs in association with profound meningeal inflammation (Kutzeinigg et al. 2005). Soluble inflammatory factors from these cells can diffuse into the cortical tissue and destroy myelin. Another presented argument for the independence of neurodegeneration from inflammation is that treatments targeting inflammation are only effective in relapsing type of the disease, being largely ineffective in progressive forms of MS. Possible explanation of that will be discussed later in this paper.

Inflammation in multiple sclerosis appears as a crucial multi-step process beginning with peripheral immune reactions creating autoreactive T cells, transmigration of immune cells through blood–brain barrier, followed by demyelination, degeneration and axonal damage in the white and gray matter.

## Peripheral inflammatory reactions

The exact factors that initiate inflammation in MS are unknown, but it is generally accepted that environmental factors play an important role in genetically susceptible individuals triggering T cell mediated response against CNS. Myelin-specific activated autoreactive T cells are found in the peripheral blood of MS patients. Molecular mimicry mechanism in which T cells generated against non self, viral or microbial epitopes, cross react with self myelin epitopes of the similar sequence has been postulated as a potential autoimmune mechanism (Comabella and Khoury 2012). There are several genes that confer susceptibility to MS. Among the most important are genes of the major histocompatibility complex like HLADRB1*1501 allele, HLA DQA1*0102 allele or HLA DQB1*0602 allele, responsible for about 50 % genetic risk for MS. It has also been found that HLA DRB1 1501 allele is linked to disease severity. Other genes, which have been identified code for receptors of interleukin 2 and interleukin 7.

## Migration of inflammatory cells to the CNS

The leukocyte trafficking to the brain of MS patients is a complex process (Rubenko-Moll et al. 2006). It is initiated by tethering and rolling of the leukocytes on endothelial surfaces, mediated by endothelial selectins which interact with glycosylated ligands on the leukocytes. The L, E, and P selectins participate in this process.

A significant increase in soluble forms of selectins has been found in the sera of patients with relapsing–remitting MS and correlated with disease activity (Losy 1999; Kuenz et al. 2005).

The rolling leukocytes interact with chemokines, which are immobilized on endothelium, and bind to their receptors on leukocytes. The chemokine receptors activation results in G-protein signal and the activation of leukocyte integrins changing their state from low to high affinity/avidity. The activated integrins interact with their endothelial counter-receptors of the immunoglobulin superfamily. Among the most important are: the β2 integrin leukocyte function associated antigen (LFA-1) binding to ICAM-1 and the α4β1 integrin very late antigen-4 (VLA-4) binding to VCAM-1. These interactions result in leukocyte arrest and adhesion on the endothelial surface. Increased soluble ICAM-1 levels have been found in the CSF and sera of MS patients and correlated with disease activity (Hartung et al. 1993). Elevated concentrations of sVCAM-1 in the CSF and sera have also been found in MS patients (Droogan and McMillan 1996). The correlation between sVCAM-1 and gadolinium enhancement in MRI has been shown (Hartung et al. 1995).

Another important adhesion molecule is the platelet endothelial cell adhesion molecule-1 (PECAM-1) (Woodfin et al. 2007). The expression of PECAM-1 on endothelial cells is concentrated at cell to cell junctions. PECAM-1 binds to itself and also to leukocyte αvβ3 integrin, an adhesion molecule found on endothelial cells and NK cells. The process of migration through intercellular junctions of vascular endothelial cells is mediated by homophilic adhesive interactions that take place between leukocyte and endothelial cell junctional PECAM-1 molecules. Increased levels of sPECAM-1 have been detected in serum and CSF of patients with active, gadolinium enhancing MS lesions on MRI (Losy et al. 1999).

Following leukocyte arrest, in the locomotion phase of the process the leukocytes travel across endothelial surfaces in search of interendothelial junctions. After protrusion, transmigration occurs in response to the abluminal chemokines according to the chemotactic gradient. Next, the leukocytes penetrate across the endothelial basement membrane to the perivascular space. Entry into the brain parenchyma needs a transversing of the glia limitans and its associated basement membrane which requires the action of matrix matalloproteases. The association between specific MMP serum levels, clinical and MRI activity has been observed in MS patients (Helgeland and Gilhus 2012).

Different pairs of chemokine receptors and their ligands play a pathogenic role in MS. Chemokines stimulate integrin activation, mediate leukocyte locomotion on endothelial cells and participate in transendothelial migration according to chemoattractant gradients. The CXCR3 receptor is expressed on the majority of T cells in the CSF of patients with MS. CXCL9 and CXCL10, CXCR3 receptor ligands were elevated in the CSF of patients with MS during relapse. These chemokines were also detected in actively demyelinating lesions. CXCL12 and CXCL13 are crucial for B cell trafficking to the CNS. Both chemokines have been found to be elevated in the CSF of patients with MS and demyelinating lesions (Krumbholtz et al. 2006). CX3CR1 participates in NK cell migration to the CNS. It has been shown that these cells correlate with disease activity in MS patients (Infante-Duarte et al. 2005).

## Involvement of inflammatory cells in demyelination and neurodegeneration

Inflammatory pathogenic T cells, which enter the CNS initiate a complex immunological cascade consisting of epitope spreading, which triggers new attacks and activation of the innate immune system consisting of microglia, dendritic cells, astrocytes and B cells which leads to chronic CNS inflammation (Weiner 2009).

Infiltrating cells secrete proinflammatory cytokines like IFN-γ, TNF-α, IL-2. Another important cytokine playing a significant role in MS is IL-18, stimulating the release of IFN-γ (Losy and Niezgoda 2001). Proinflammatory cytokines stimulate the release of chemokines, expression of adhesion molecules and can be factors damaging both myelin sheath and axons. Th2 CD4+ cells release anti-inflammatory cytokines having an immunomodulatory effect. The regulatory T cells (CD4+ CD25+ Treg) are a small subset of CD4+ T cells. Defects in functional suppression by these cells in MS patients has been described (Viglietta et al. 2004).

Th17 cells are also participating in MS neuroinflammation. These cells release the proinflammatory cytokine IL-17. It has been shown that IL 23 produced by macrophages and dendritic cells is important for the expansion of Th17 cells (Korn et al. 2009). Th 17 cells in the CSF of MS patients are significantly increased during exacerbations in comparison with remission periods (Brucklacher-Waldert et al. 2009).

CD8+ cells are also involved in inflammatory reaction and correlate better with axon destruction in demyelinating lesions than CD4+ cells (Bitsch et al. 2002). CD8+ cells can directly damage axons or this damage can follow the destruction of the myelin sheath and oligodendrocytes (Melzer et al. 2009).These cells may transect neuritis in an MHC class I/peptide-restricted fashion. The mechanism of axonal damage is multifactorial and includes also action of proteases and free radicals released during CNS inflammation as well as lack of neurotrophic factors provided to axons (Comabella and Khoury 2012). B cells stimulated by IL-4, IL-6 and IL-10 are involved in the production of antibodies which can participate in demyelination in the form of immune complexes which activate complement or participate in antibody dependent cell cytotoxicity. B cells have also the capacity to function as antigen presenting cells and contribute to T cell activation (Barun and Bar-Or 2012).

Neuroinflammation is not only present in relapsing–remitting multiple sclerosis but also in the secondary and primary progressive forms of the disease. T and B cell infiltrates correlate well with the activity of demyelinating lesions, while plasma cells seem to be most pronounced in patients with secondary and primary progressive MS. A highly significant association between inflammation consisting of T cells, B cells, plasma cells and macrophages and axonal injury exists in MS patients including progressive forms of the disease alone (Frischer et al. 2009).

## The effect of immunomodulating, anti-inflammatory therapies

Anti-inflammatory therapies with a different mode of action influence the disease course. Natalizumab which is a monoclonal antibody against VLA-4 integrin, interfering with the process of leukocytes adhesion to endothelium, reduces significantly the relapse rate, the risk of disability progression and MRI activity (Polman et al. 2006).

Alemtuzumab causing prolonged depletion of peripheral blood lymphocytes shows better results in comparison with IFN-β 1a in the reduction of the relapse rate and the risk for sustained disability.

Rituximab a chimeric antibody against CD20 on B cells has shown a reduction in MRI activity and a reduction of the relapse rate (Hauser et al. 2009). Another monoclonal antibody, daclizumab, directed against the α subunit (CD25) of IL-2 receptor inhibits activated T cells. It can also cause an expansion of the subset of NK cells, CD56 bright cells, which lyse autologous T cells. Added to treatment with interferon β 1a reduced significantly the MRI activity of MS patients (Wynn et al. 2010).

Several oral new dugs have also anti-inflammatory properties and affect clinical and MRI activity in MS patients. Fingolimod, sphingosine-1-phosphate receptor modulator reversibly sequesters lymphocytes mainly in the lymph nodes, reducing their recirculation to the CNS and abrogating the neuroinflammatory process (Kappos et al. 2006). It has been also shown to suppress the generation of Th17 cells in vitro (Liao et al. 2007). Triflunomide blocks lymphocyte T and B proliferation by inhibiting dehydroorotate dehydrogenase (DHODH), a key enzyme needed for pyrimidine synthesis (O´ Connor et al. 2006). Dimethyl fumarate (BG-12) induces a shift from Th1 (proinflammatory) to Th2 (anti-inflammatory) cytokine response. It decreases also the expression of ICAM-1 and VCAM-1 (De-Hyung Lee et al. 2008). It is now established that anti-inflammatory and immunomodulatory treatment is beneficial in the early relapsing stage of MS, but these treatments are ineffective in secondary progressive and particularly in primary progressive MS. Is this a proof for the independence of neurodegeneration from the inflammatory process? Not necessarily. In the stage of progressive MS, inflammation becomes trapped behind a closed or repaired blood–brain barrier (Lassmann 2010). In such a situation current immunomodulatory, immunosuppressive or anti-inflammatory treatments might not reach this inflammatory process in concentrations which are sufficient to exert a beneficial effect. Neurodegeneration caused by inflammation in earlier stages of the disease is dominant in later stages of MS and requires neuroprotective and neuroregeneration treatment strategies.
